# Computational modelling of cell chain migration reveals mechanisms that sustain follow-the-leader behaviour

**DOI:** 10.1098/rsif.2011.0726

**Published:** 2012-01-04

**Authors:** Michelle L. Wynn, Paul M. Kulesa, Santiago Schnell

**Affiliations:** 1 Department of Molecular and Integrative Physiology and Center for Computational Medicine and Bioinformatics, University of Michigan Medical School, Ann Arbor, MI 48109, USA; 2 Stowers Institute for Medical Research, Kansas City, MO 64110, USA; 3 Department of Anatomy and Cell Biology, University of Kansas School of Medicine, Kansas City, KS 66160, USA

**Keywords:** developmental biology, cell migration, chain migration, neural crest cells, agent-based modelling, computational biology

## Abstract

Follow-the-leader chain migration is a striking cell migratory behaviour observed during vertebrate development, adult neurogenesis and cancer metastasis. Although cell–cell contact and extracellular matrix (ECM) cues have been proposed to promote this phenomenon, mechanisms that underlie chain migration persistence remain unclear. Here, we developed a quantitative agent-based modelling framework to test mechanistic hypotheses of chain migration persistence. We defined chain migration and its persistence based on evidence from the highly migratory neural crest model system, where cells within a chain extend and retract filopodia in short-lived cell contacts and move together as a collective. In our agent-based simulations, we began with a set of agents arranged as a chain and systematically probed the influence of model parameters to identify factors critical to the maintenance of the chain migration pattern. We discovered that chain migration persistence requires a high degree of directional bias in both lead and follower cells towards the target. Chain migration persistence was also promoted when lead cells maintained cell contact with followers, but not vice-versa. Finally, providing a path of least resistance in the ECM was not sufficient alone to drive chain persistence. Our results indicate that chain migration persistence depends on the interplay of directional cell movement and biased cell–cell contact.

## 1. Introduction

Long-distance cell migration involves directed and sustained cell movements that produce an ordered invasion of target sites. A striking cell migratory behaviour observed in a wide variety of embryonic and adult model systems is follow-the-leader chain migration [1–3], where cells travel in loosely connected linear arrays. Imaging advances now permit the visualization of cell migratory behaviours in tissue slices, three-dimensional arrays, the living embryo and adult animal systems. While our understanding of cell migration has advanced, little is known about the mechanisms that drive follow-the-leader chain migration.

Experimental evidence suggests that chain migration in adult neurogenesis [2] and cancer metastasis [3,4] is regulated by multiple cellular and molecular cues, the coordination of which at the single-cell level is still unclear [5–9]. Chain migration also occurs in vertebrate embryonic development where disruptions in normal developmental migration programmes are associated with a number of developmental disorders. Identification of the mechanisms that sustain chain migration will have important implications for a number of cell invasive systems.

In vertebrate embryonic development neural crest (NC) cells [10] delaminate from the dorsal neural tube and are sculpted onto stereotypical migratory pathways in a head-to-tail manner [11]. NC cell chain migration has been reported in a number of model systems including chick, zebrafish and mouse [11–14]. NC cell chains in living chick embryos have been observed to persist *in vivo* for at least 10–15 h over distances up to 600 µm. These chain-like arrays of cells have been visualized at nearly all axial levels, including the head, cardiac and trunk [15–17]. Typically, NC cell chain-like arrays are made of loosely connected cells, which have dynamic filopodial contacts between the cells that are sustained, broken and re-established during chain migration [15,16].

It is likely that a variety of spatio-temporal factors are involved in the primary mechanism of chain migration. Because it will be difficult for experimental data alone to provide an integrative view of such a complex mechanism, theoretical modelling can help isolate critical factors and direct future experiments. Owing to limited quantitative information, it is difficult to construct a purely mathematical model of chain migration, however. An agent-based model (ABM), on the other hand, is a powerful computational tool for investigating complex phenomena that are not readily explored by experimental or mathematical methods [18].

Most mechanistic hypotheses of cell migration have focused on the establishment of directional migration to form and maintain discrete cell migratory streams [19–23]. We developed an ABM to explicitly test two mechanistic hypotheses of follow-the-leader chain migration using the NC as a model system: (i) trailing cells follow a path of lesser resistance within the extracellular matrix (ECM), forged by a lead cell and (ii) contact guidance by cell–cell filopodial interactions direct trailing cells to follow a lead cell.

## 2. Material and methods

### 2.1. Integration of experimental data

**Figure 1.**
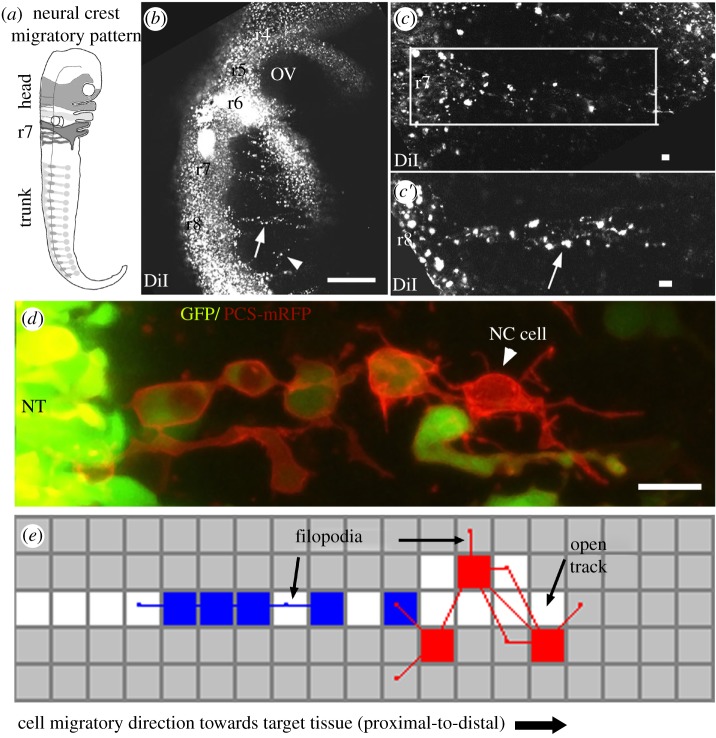
Neural crest (NC) cell chain migration in development and computational model schematic. (*a*) The NC cell migratory pattern is comprised of migratory streams and chain-like arrays that stretch from the dorsal neural tube to the periphery throughout the head and trunk. (*b*) *In vivo* imaging in the living chick embryo taken from a typical confocal imaging session in which the lipophilic dye, DiI, has been injected into the lumen of the neural tube to label premigratory NC cells that migrate into surrounding unlabelled tissue. (*c*–*c*′) NC cell chain migration is shown at higher magnification in DiI-labelled NC cells. (*d*) NC cells labelled with green fluorescent protein and a membrane-specific fluorescent reporter (PCS-mRFP) highlight the distinct *in vivo* features of NC cell chain migration, observed in a typical confocal time-lapse imaging session. (*e*) Snap-shop of a Contact Model simulation. An *in silico* framework was used to test mechanistic chain migration hypotheses. The framework assumes chain migration occurs on a two-dimensional grid with time split into discrete steps. The migratory target is the distal edge of the grid. Two types of agents (cells) are used in the simulations: Hairy *Leader* agents near the front of the chain with many protrusions and polarized *Follower* agents near the back of the chain with fewer protrusions. After an agent has moved to a site on the grid, the site becomes Open, possibly leading to open tracks of least resistance in the simulation domain. An agent may move in one of the eight directions (determined by the sites immediately adjacent to the agent) according to a set of rules and parameters that govern how individual agents move each time step. Scale bars, (*b*) 100 µm and (*c*,*d*) 10 µm. Rhombomere, r; neural crest, NC; neural tube, NT. Grey regions, Closed site; white regions, Open site; blue regions, *Follower* cell; red regions, *Leader* cell.

The NC is an excellent model system for the integration of theory and experiment owing to an extensive literature [10] and exciting emerging data [11–14,24,25]. NC cell chain migration is accessible to fluorescent cell labelling and *in vivo* imaging in several model systems (figure 1*a*–*d*). In the chick embryo, typical NC cell chain migration occurs from the dorsal neural tube into the periphery and appears to involve contact between neighbouring cells (figure 1*d*). Several key experimental observations of NC cell chain migration considered during model development are summarized in table 1.

**Table 1.** Key observations of *in vivo* dorsal neural tube chain and cell migration considered during model development.

observation	reference	use in the model
while migrating from the neural crest to target destinations, many cells in sub-populations of neural crest cells form linear chain structures with a clear Leader cell	[16,17,26]	treated as model assumption
cells can join or leave a chain during migration and are not restricted to a chain	[15,27]	treated as model assumption
chains disassemble laterally from the neural tube	[16]	treated as model assumption
chains stretch approximately 180 µm in length and are typically six cells long and two cells wide	[17]	treated as model assumption
cells near the front of the chain tend to be hairy with many protrusions while trailing cells tend to have only a few protrusions	[26]	treated as model assumption
the maximum protrusion length between linked cells is approximately 100 µm	[16,17]	treated as model assumption
cells moving in chains tend to move faster and with more directionality than individual cells	[16]	treated as model assumption
cells in a chain extend and retract filopodia in short-lived cell contacts that are sustained, broken and re-established	[15,16]	treated as model assumption
trailing cells tend to be polarized in the direction of migration	[26]	model parameter SID (Synchronize Initial Directions)
filopodia have sometimes been observed to lengthen or shorten between two linked cells as one cell moves away or towards the other	[26,28]	model parameters FMP and LMP (Follower/Leader Maintain Protrusions)
cells have been observed changing direction in order to move towards filipodial contacts with other cells; contact inhibition of locomotion has also been reported	[26,28]	model parameters FMC and LMC (Follower/Leader Move towards Contact)
filopodia have sometimes been observed to retract after a cell moves towards a contacted cell	[26]	model parameters FRP and LRP (Follower/Leader Retract Protrusions)

### 2.2. Agent-based model formulation

An agent-based modelling framework was developed to test two distinct mechanistic models. In an ABM, a system is modelled as a collection of autonomous decision-making entities called agents that execute behaviours according to a set of rules defined from empirical or experimental observations of the phenomenon under investigation. There were two types of agents in our models: *Leaders* and *Followers*. Each simulation consisted of three *Leaders* and five *Followers* that could move in any of eight directions (figure 1*e*).

**Figure 2.**
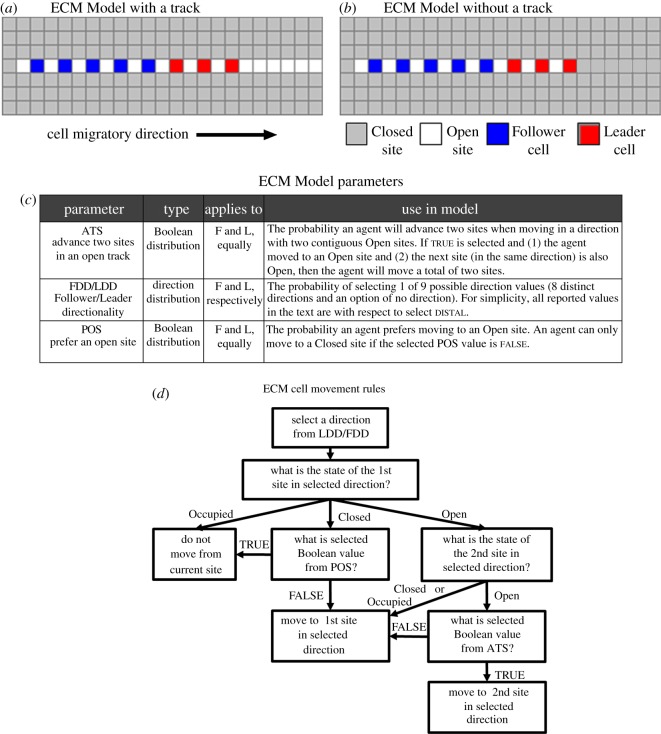
The ECM Model schematic, parameters and cell movement rules. Two types of ECM Models were tested: (*a*) one with an existing track and (*b*) one without an existing track in the simulation domain. Existing tracks are represented as contiguous Open (white) sites on the grid and are assumed to have been left by a preceding cell (or cells). Spatial arrangements shown are the initial configurations (at time = 0) used in each ECM Model type. (*c*) There were four parameters in the ECM Model that, along with a set of rules, controlled how agents moved in the simulations. F and L refer to *Follower* and *Leader* agents, respectively. (*d*) A summary of the rules that executed each time a *Leader* or *Follower* was selected in the simulation is presented. The complete rule algorithm is provided in the electronic supplementary material, §2.1.

Sites on the simulation grid could be in one of three possible states: (i) Closed—no agent has occupied the site, (ii) Open—an agent occupied the site but left, and (iii) Occupied—an agent currently occupies the site. Whenever an Occupied site was selected, the agent it contained was updated according to a specific set of rules. The rule algorithms developed for the ECM and Contact Models were based on experimental data (table 1) and were designed to test the underlying mechanistic hypothesis of each model. The algorithms are provided in the electronic supplementary material, §2 and summarized in figures 2*d* and 4*b*. A description of how the rules were applied in each model is also provided in §3.

Both models assumed cell movement occurred on a two-dimensional rectangular grid with non-cyclic boundary conditions and that the distal edge of the grid was the migratory target. The simulation grid consisted of 6750 sites with 150 sites along the *x*-axis (proximal-to-distal) and 45 sites along the *y*-axis (anterior-to-posterior). The width of each site was assumed to be equivalent to 30 µm (approx. equal to the reported average length of a single NC cell in a chain with extended protrusions) [17]. We did not explicitly define a timescale *a priori* because the interval between successive time steps in an ABM has arbitrary physical units. However, because the models simulated coarse-grained movements in increments of 30 µm, we assume a time step is roughly on the order of hours.

### 2.3. Definition of a chain in the model

In order to simulate chain persistence in our models, we developed a computational definition of a chain that was based on published reports of chain structure [17] and average speed [16]. NC chain migration is a dynamical system where cells within a chain extend and retract filopodia in short-lived cell contacts that are sustained, broken and re-established [15,16]. In our models, a chain was required to contain at least six sequential agents that moved together towards the target by a minimum velocity. Two agents were considered sequential if there were no empty sites between the two agents along the anterior-to-posterior axis (*y*-axis) and no more than three empty sites between the two agents along the distal-to-proximal axis (*x*-axis). The minimum velocity was defined as a collective displacement towards the target of one site every 10th time step (electronic supplementary material, §1).

### 2.4. Determination of the factors affecting chain migration

Our analyses began with a pre-defined pattern (a chain of cells) and consisted of the quantitative evaluation of parameters most critical for the maintenance of the pattern. This approach is a departure from classical ABM analysis, which is generally interested in investigating qualitative emergent patterns. We used chain persistence (defined as the number of time steps agents travelled as a chain during a simulation) as a sensitivity measure to quantify how different parameters affected chain stability over time. If the model operated in a parametrically sensitive region, we expected the migratory pattern to break with small variations in parameter values. Specifically, we examined the influence of a parameter by systematically varying the parameter and comparing the mean chain persistence after 400 repeats to that of a baseline parameter set prior to a variation. It was necessary to repeat simulations many times for the same parameter set to obtain an average persistence because of the stochastic nature of the models developed (electronic supplementary material, §1). While our method was specifically designed to identify parameters that affect chain persistence, an alternate sensitivity approach for ABMs is global uncertainty analysis [29].

In our analyses, three types of parameters were varied: (i) integer values, (ii) Boolean values (true or false), and (iii) probability distributions. Probability distributions consisted of a set of possible values (either directional or Boolean) where each value had an associated probability to be selected. An example of a Boolean probability distribution is true = 25% and false = 75%. The finite set of values in a directional distribution consisted of eight possible directions as well as the value of none (if selected, no directional decision would be made). A reported directional probability of 11 per cent (or approx. 1/9) represents the condition where all direction values in the distribution are equally likely.

### 2.5. Computational implementation and statistical analysis

The ABM framework was implemented in Java (Sun Java JDK v. 1.6.0_20) and simulations were performed on Intel Nehalem/i7 Core processors running Red Hat Linux. At the end of each simulation, the chain persistence was recorded. For each parameter set tested, simulations were repeated 400 times and a mean chain persistence ± the standard error of the mean (s.e.m.) was reported. To assess significance of a mean persistence value after a parameter change, the Tukey–Kramer method was used to evaluate pairwise comparisons with a baseline persistence at a significance level of 95 per cent (*α* = 0.05). Pearson's product–moment correlation [30] was used to measure the linear association between parameter values. Reported *p*-values for correlations refer to the probability that the true correlation is equal to 0.

## 3. Results

All simulations began with three *Leader* and five *Follower* agents initialized on the grid to mimic embryonic NC cell chain migration (figure 1). There were fewer *Leaders* than *Followers* in the simulations because time-lapse imaging in chick shows that stereotypic NC cell chains consist of a few leading hairy cells followed by several polarized cells [26]. Default chain migration persistence baselines were established for each model. To limit parameter bias, values in a default parameter set were chosen so that all probability distribution values were equally likely, discrete numeric values equalled 5, and discrete Boolean values equalled false. To denote the probability of selecting a given value from a parameter's probability distribution, the following general notation is used: PARAMETER-value. For example, the probability of selecting false or distal from the Prefer an Open Site (POS) or Leader Directionality Distribution (LDD) parameters, respectively, would be POS-false or LDD-distal.

### 3.1. Extracellular Matrix Model

The ECM Model was designed to test the hypothesis that a path of least resistance in the ECM can explain the persistence of migratory chains observed experimentally. NC cell tracking indicates that later emerging chick post-otic NC cells tend to migrate along trajectories of preceding cells [17]. To investigate the importance of a path opened by a preceding cell, the ECM Model was analysed in both the presence (figure 2*a*) and the absence (figure 2*b*) of a pre-existing track. If a track was included, all sites in the same row as the chain and between the first agent and the distal edge of the grid were initialized with a state of Open.

#### 3.1.1. Description of movement rules in the Extracellular Matrix Model

Four ECM Model parameters (figure 2*c*), along with a set of rules (figure 2*d*), controlled an agent's movement in the simulation domain. When an agent was selected for an update in the simulation, it first chose a value from the LDD or Follower Directionality Distribution (FDD) probability distributions (depending on whether it was a *Leader* or *Follower*, respectively). If none was selected, the agent would remain stationary for this time step. Otherwise, the agent would evaluate whether the first adjacent site in the chosen direction was Open, Closed or Occupied. If the site was Closed, the agent would select a value from the POS distribution and would only exert the force needed to forge into the Closed region of the ECM, if false was selected from POS. If the selected site was Open, however, the agent would move to that site and if the next site in that direction was also Open, the agent would select a value from the Advance Two Sites in Open Track (ATS) distribution. If true was selected from ATS, the agent would then move one additional space (effectively moving faster as a result of an Open channel in the ECM).

#### 3.1.2. Determination of high persistence parameter sets in the Extracellular Matrix Model

**Figure 3.**
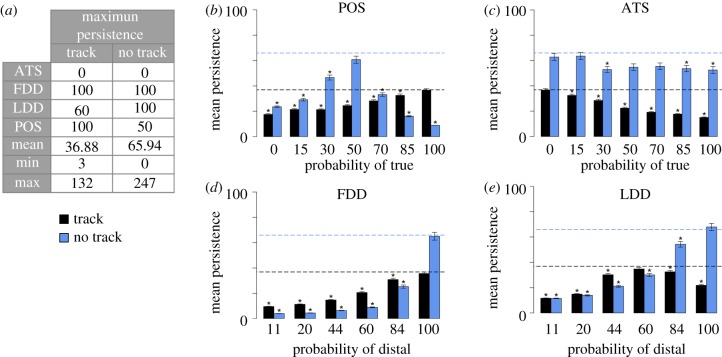
Analysis of maximum persistence levels in both ECM Model types. (*a*) The maximum mean chain migration persistence with and without an existing track was 36.88 ± 1.20 and 65.94 ± 3.10 time steps, respectively. The parameter sets that produced the maximum persistence in each model type represent the Track and No Track maximum persistence baselines, respectively (see electronic supplementary material, movie S3 and S4, respectively, for representative simulations under these conditions). The maximum baselines were identified by testing all 6400 possible parameter sets with and without a track. The specific parameter values, mean chain migration persistence and the minimum (min) and maximum (max) persistence observed in 400 simulations are listed for each baseline's parameter set. The presented parameter values are the per cent probability to select true and distal from the Boolean (ATS and POS) and directionality (LDD and FDD) distributions, respectively. (*b*–*e*) Parameter sensitivity to the maximum baselines was tested by systematically varying one parameter while the other three parameters were held fixed to their corresponding baseline value (listed in the table). The results of perturbing each of the four parameters are presented for both model types. The mean chain migration persistence of the maximum baselines with and without a track is represented as black and blue dashed lines, respectively. Mean persistence values statistically different from the associated baseline are indicated by asterisk (*) (*α* = 0.05). All mean persistence values were calculated from 400 simulations with the same parameter set. Error bars reflect the s.e.m. (*b*–*e*) Black regions, track; blue regions, no track.

The mean chain migration persistence of the default baseline in the ECM Model was 5.56 ± 0.14 and 5.39 ± 0.15 time steps, respectively, with and without a pre-existing track (electronic supplementary material, table S1 and movies S1–S2). To identify conditions that contribute to high chain migration persistence, 6400 distinct parameter sets were generated using a finite set of possible distribution values for the four parameters (electronic supplementary material, §3). For both ECM Model types, the majority of the 6400 parameter sets produced mean chain migration persistence of 10 or fewer time steps (electronic supplementary material, figure S1*a,b*). The maximum mean chain migration persistence, with and without an existing track, was 36.88 ± 1.20 and 65.94 ± 3.10 time steps, respectively (figure 3*a* and electronic supplementary material, movies S3–S4). These parameter sets are referred to as the Track and No Track maximum persistence baselines, respectively. To better understand the importance of individual parameters at high persistence levels, we systematically varied a single parameter value in the maximum persistence baselines while keeping the remaining three parameters fixed to their baseline values.

#### 3.1.3. High chain migration persistence in the Extracellular Matrix Model required *Leader* and *Follower* agent directional bias

In the absence of an existing track, chains persisted longest when *Leaders* and *Follower* were completely biased to select the target direction (i.e. LDD*-*distal and FDD-distal = 100%). In the presence of an existing track, however, chains persisted longest when FDD-distal = 100% but LDD-distal = 60% (figure 3*d*,*e*). In qualitative observations of ECM Model simulations with a track, when LDD*-*distal = 100%, *Leaders* appeared to move too rapidly for the other agents to keep up, resulting in more rapid chain disassembly (electronic supplementary material, movie S5). Together, these results suggest that a modulation in *Leader* velocity towards the target favoured chain migration persistence if a track was already present in the environment. Otherwise, in the absence of an existing track, complete directional bias by both types of agents was needed.

The importance of specific parameter probability values in the ECM Model can be seen in frequency distributions of the parameter sets that produced high mean chain migration persistence (electronic supplementary material, figure S1*c–f*). The majority of parameter sets producing mean chain migration persistence of 20 time steps or more had an FDD-distal probability of at least 84 per cent and an LDD-distal probability of at least 60 per cent (electronic supplementary material, figure S1*e*–*f*). These data indicate a need for very high directional bias by *Followers* in the ECM Model and suggest that a path of least resistance was not enough, by itself, to direct *Follower* to follow *Leaders* in high persisting chains.

An agent could only proceed to a selected Closed site if false was selected from the POS distribution (figure 2*d*). POS, therefore, controlled the probability that an agent would be willing to exert the force needed to move into unopened areas in the simulation domain. When an existing track was present, mean chain migration persistence was highest when agents never moved into Closed areas (POS*-*true = 100%). In contrast, when no existing track was present, chain migration persistence was highest when POS*-*true = 50% and lowest when POS*-*true = 100% (figure 3*b*). These data suggest that, in order to maintain high persistence when no existing track was available, individual agents needed the ability to explore unopened regions, which would not be permitted if POS-true = 100%.

The default behaviour in all simulations was for agents to advance one site each time step. The ATS parameter controlled the probability that any agent could instead advance two sites if moving towards two contiguous Open sites (figure 2*d*), conditionally moving faster when true was selected from ATS. In general, mean chain migration persistence decreased as ATS-true increased (figure 3*c*). This effect was most pronounced when an existing track was present in the simulation domain. These results suggest an agent's ability to move faster in a path of least resistance did not promote chain migration persistence in our models.

### 3.2. Contact Model

**Figure 4.**
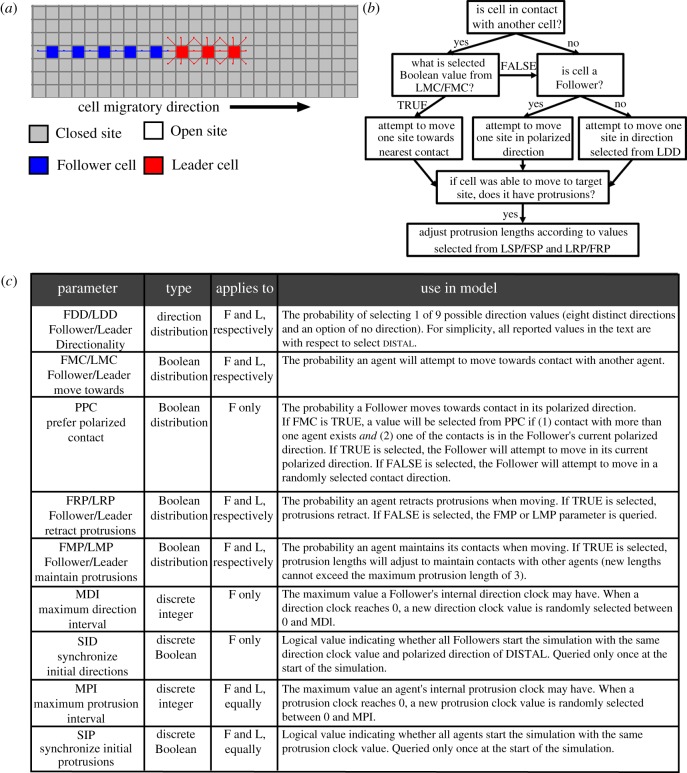
The Contact Model schematic, parameters and cell movement rules. (*a*) Agents in the Contact Model had filopodia that extended and retracted according to a set of rules. (*b*) A summary of the rules that executed each time a *Leader* or *Follower* agent was selected in the simulation is presented. (*c*) There were 13 model parameters in the Contact Model that, along with the set of rules, controlled how agents moved in the simulations. The FMC, LMC, PPC, FRP, LRP, FMP and LMP parameters controlled protrusion-related behaviours. F and L refer to *Follower* and *Leader* agents, respectively. The complete rule algorithm is provided in the electronic supplementary material, §2.2.

The Contact Model was designed to test the hypothesis that filopodial contact can explain the maintenance of migratory chains observed experimentally. The initial basis of this hypothesis is the assumption that lead cells are somehow directionally biased while the remaining cells in the chain follow by responding to contact guidance cues from the filopodia of cells around them. A variety of NC cell behaviours associated with cellular protrusions have been reported *in vivo* (table 1). Because the frequency of many of these behaviours has not been experimentally measured, they have been included as probability distribution parameters in the model (figure 4*c*).

In the Contact Model, each *Follower* had a polarized direction that allowed for the inclusion of persistence of direction for the polarized phenotype. Because we do not know precisely the combination of signals that induce a cell to change direction or to extend or retract filopodia, these events were stochastically influenced by internal clocks unique to each agent. Two types of clocks were used: a polarized direction change clock and a protrusion change clock. Clocks were implemented as counters that decremented each time step. When a *Follower*'s polarized direction change clock reached 0, the *Follower* selected a new polarized direction from the FDD probability distribution. Similarly, when a Follower or *Leader* agent's protrusion change clock reached 0, the agent toggled its current protrusion state (by retraction or extension of protrusions). Direction or protrusion change clocks randomly picked a new value between 0 and the Maximum Direction Interval (MDI) or Maximum Protrusion Interval (MPI) parameter, respectively, after reaching 0.

#### 3.2.1. Description of movement rules in the Contact Model

Thirteen Contact Model parameters (figure 4*c*), along with a set of rules (figure 4*b*), controlled an agent's movement in the simulation domain. Agents in the Contact Model were initialized in the same configuration as the ECM Model (figure 2*a*,*b*), except with extended protrusions (figure 4*a*). The rule algorithm used in the Contact Model allowed agents to conditionally move in response to contact with other agents and accounted for extension and retraction of cellular protrusions. The following general rules applied to protrusions in the Contact Model: (i) *Leaders* were hairy with up to eight protrusions, (ii) *Followers* were polarized with up to two protrusions oriented along the direction of movement and (iii) protrusions could not exceed three sites in length (which is equivalent to the approximately 100 µm reported as the maximum protrusion length observed experimentally [16,17]).

Before an agent moved in the Contact Model, it first determined if it was in contact with another agent based on whether its filopodia were in contact with another agent or if another agent's filopodia were touching the selected agent. If contact with another agent existed and true was selected from Leader Move Towards Contact (LMC) or Follower Move Towards Contact (FMC) distribution, respectively, the *Leader* or *Follower* agent attempted to move one space in the direction of contact. If a *Leader* did not move as a result of contact with another agent, either because it did not receive a signal to do so (because no contact existed) or it did not respond positively to the signal (because false was selected from LMC), the *Leader* would attempt to move in a direction selected from LDD. If a *Follower* did not move as a result of contact with another agent, it would attempt to move one space in its current polarized direction.

#### 3.2.2. Determination of high persistence parameter sets in the Contact Model

The mean chain migration persistence of the default baseline in the Contact Model was 3.27 ± 0.11 time steps (electronic supplementary material, table S2 and movie S6). To identify conditions that contribute to high chain migration persistence in the Contact Model, we analysed randomly selected parameter sets. The same granularity of parameter values used to produce 6400 distinct parameter sets in the ECM Model would generate 2.0 × 10^9^ distinct parameter sets in the Contact Model. Because testing such a large number of parameter sets was not computationally practical, we randomly selected 100 000 parameter sets (electronic supplementary material, §3). Only 488 (approx. 0.5%) of the randomly selected parameter sets produced mean chain migration persistence of 20 or more time steps (electronic supplementary material, figure S2*a*). We refer to this subset as the high-persistence sub-group.

**Figure 5.**
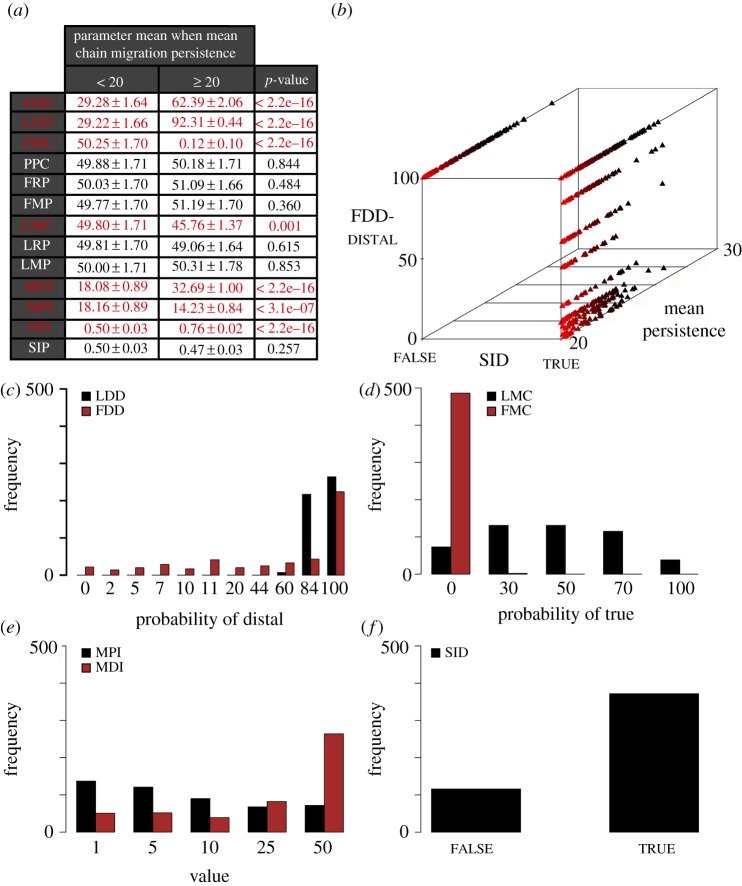
Seven Contact Model parameters contributed to high mean chain migration. One hundred thousand parameter sets were randomly selected in the Contact Model. (*a*) Only 488 randomly selected parameter sets produced mean chain persistence ≥ 20 time steps. The mean parameter values in this group (middle column, ≥20) were compared with the mean parameter values in the parameters sets that produced mean chain migration persistence < 20 time steps (*n* = 99 512, left column, <20). Reported values are the mean parameter value ± s.e.m. Parameters highlighted in red have *p*-values ≤ 0.001. Boolean SID and SIP values were converted to 0 and 1 for this analysis. (*b*) A striking correlation in the data was the relationship between FDD-distal probabilities, true or false SID values, and mean chain migration persistence. When SID was true, a range of FDD-distal values was observed but, when SID was false, only FDD-distal = 100% was observed. (*c*–*f*) The parameters with statistically different means in the two groups had non-uniform frequency distributions in the set that produced mean chain migration persistence ≥ 20 time steps (compare with electronic supplementary material, figure S2*c*–*f*).

To understand the specific parameter conditions that contributed to high chain migration persistence, we determined the mean value and frequency of each parameter value in the high-persistence sub-group. In general, if persistence was sensitive to a particular parameter, we expected the parameter to have a mean value in the high-persistence sub-group that differed significantly from its mean in a random sample of parameter sets. Comparisons of parameter means in the high-persistence sub-group to parameter means in the remaining randomly selected parameter sets (*n* = 99 512), revealed that seven parameters had statistically different means in the two groups (figure 5*a*). These same parameters exhibited non-uniform value distributions in the high-persistence sub-group (figure 5*c*–*f*), while the remaining six parameters exhibited mostly uniform value distributions in the high-persistence sub-group (electronic supplementary material, figure S2*c*–*f*).

#### 3.2.3. High chain migration persistence in the Contact Model required high *Leader*, but not necessarily high *Follower*, directional bias

It is clear from histograms of parameter values that *Leaders* were highly biased to select distal from LDD when persistence was highest (figure 5*c*). In the high-persistence sub-group, nearly all of the parameter sets (98.6%) had an LDD-DISTAL value ≥84%. This result suggests that high chain migration persistence was favoured when *Leaders* were most likely to select the distal direction if they did not first move as a result of contact with another agent. In contrast, while FDD-distal = 100% favoured persistence, more than a third (38.5%) of the parameter sets in this group had an FDD-distal probability less than or equal to 44 per cent (figure 5*c*). This result suggests a mechanism other than the sensing of external stimuli (approximated as high FDD-distal probabilities) may have played a role in guiding *Followers* at high persistence levels.

#### 3.2.4. Leaders appeared more reliant on contact-guidance than *Followers* in high persisting chains

In the high-persistence sub-group, chain migration persistence was highest at specific values of the discrete MDI and MPI parameters (figure 5*e*). As described above, each *Leader* and *Follower* had its own protrusion change clock and each *Follower* had its own direction change clock. Clocks decremented each time step and, upon reaching 0, a direction or protrusion change clock randomly picked a new value between 0 and the MDI or MPI parameter, respectively. The maximum value allowed for MDI or MPI was 50. A low MPI value would result in more frequent signals to retract and re-protrude, and a high MPI value would result in protrusions that were more likely to stay extended or retracted for many time steps. More than half of the parameter sets in the high-persistence sub-group (53%) had MPI ≤ 5 (figure 5*e*). MPI is assumed to have the largest impact on *Leader* agents because *Followers* rarely relied on filopodia to make movement decisions in the high-persistence sub-group (figure 5*d*). These data suggest that chain migration persistence was highest when *Leaders* were more likely to probe their environment by retracting and re-extending their protrusions. In addition, more than half of the parameter sets in the high-persistence sub-group (54%) had the maximum MDI value of 50 (figure 5*e*). Compared with a low MDI value, a high MDI value would result in fewer opportunities for *Followers* to select a new polarized direction. Thus, this result suggests that chains persisted longest when *Followers* did not frequently alter their polarized directions as a result of an internal signal.

In general, chains also persisted longest when *Leaders* were likely to at least sometimes move as a result of contact with another agent (30% ≤ LMC-true ≤ 70% with a mean LMC-true of 45.76% ± 1.24%). In contrast, chain migration persistence was highest when *Followers* never moved as a result of contact (FMC-true = 0% with a mean FMC-true = 0.12% ± 0.09%; figure 5*a*,*d*). Parameter perturbations using the two parameter sets that produced maximum persistence in the 100 000 sampled parameter sets also indicated that the maintenance of *Leader* protrusions contributed to high chain migration persistence (electronic supplementary material, figures S3*e*,*f*, S4*b*,*c* and §4.2.1). These results suggest that *Leaders* were far more likely to rely on contact guidance in high persisting chains than *Followers* were.

#### 3.2.5. Synchronizing the polarized directions of *Followers* contributed to higher chain migration persistence in the Contact Model

The majority of parameter sets (76.2%) in the high-persistence sub-group had discrete Boolean values of true for the Synchronize Initial Directions (SID) parameter (figure 5*f*). A true SID value meant that all *Followers* began the simulation with a polarized direction of distal and the same initial direction change clock value (figure 4*c*). Under these conditions, all *Follower* direction clocks were initially scheduled to reach 0 at the same time step. An individual *Follower* could, however, change polarized directions asynchronously from the other *Followers* upon receiving a contact-induced signal to move towards another agent. Like MDI, the SID parameter only applied to *Follower* behaviour in the simulations. Together the SID, MDI and FMC (figure 5*d*,*f*) simulation data suggest that high persistence was favoured when all *Followers* were polarized in the target direction (figure 5*f*) and did not frequently change their direction as a result of either internal (MDI) or external contact-induced (FMC) signals.

The results reported thus far provide insight into how chain migration persistence is affected by individual parameters. To examine how pairs of parameters combined to affect persistence in the Contact Model, we calculated the Pearson's correlation coefficient (*r*) for all parameter pairs in the high-persistence sub-group. The strongest correlations were found between FDD, SID and MDI (electronic supplementary material, §4.2.2). The relationship between correlated parameters and mean chain migration persistence can be visualized in three-dimensional plots. When SID was true, a range of FDD-distal probabilities contributed to high chain migration persistence levels but, when SID was false, only FDD-distal = 100% produced mean chain migration persistence greater than or equal to 20 time steps (figure 5*b*). These data suggest that the synchronization of polarity and direction clocks in *Follower* agents is likely the mechanism that compensated for low FDD**-**distal probabilities (figure 5*c*) in some of the parameter sets in the high-persistence sub-group.

### 3.3. Hybrid Extracellular Matrix–Contact Model

It is possible that aspects of both the ECM Model and Contact Model are involved in chain migration. To examine how simulated chains behaved in a hybrid ECM–Contact Model, we combined the rules of the two models such that agents made decisions on which direction to move according to the rules of the Contact Model but only moved in a selected direction according to the rules of ECM Model (electronic supplementary material, figure S5). In general, many of the parameter ranges reported as important for chain migration persistence in the ECM and Contact Models remained important in the Hybrid Model (these results are summarized in electronic supplementary material, §4.3.1).

## 4. Discussion

The objective of our computational framework was to simplify experimentally observed cell behaviours into a model capable of providing insight into the underlying mechanisms of follow-the-leader chain migration. We used ABMs and the embryonic neural crest model system because we lacked sufficient quantitative measurements for a detailed mathematical model. It is not known, for example, how likely a NC cell is to abruptly or gradually change direction or what exact signals will trigger a cell to stretch or retract its protrusions. Consequently, these behaviours were controlled by stochastic probability distributions, random timers unique to each agent and rules that allowed contact with neighbouring agents and the surrounding environment to influence movement.

To analyse the ABMs, we used a novel approach. Instead of analysing emergent patterns in a population of agents, we began simulations with a pattern (i.e. a set of agents arranged as a chain) and quantitatively looked for the factors critical to the maintenance of the pattern. This approach required us to explicitly define a migratory chain within a simulation. The definition used was dynamic and based on published reports of cell speed and chain structures *in vivo*. As long as there were always at least six agents within defined spatial constraints, agents were classified as a chain when they moved together towards the target.

The influence of model parameters was probed by systematically varying a parameter and comparing the mean chain persistence in 400 simulations to that of a baseline prior to parameter variation. Thus, mean chain migration persistence was used as a sensitivity measure that allowed us to quantify how different parameters affected chain stability over time. If chain migration in the model was sensitive to a given parameter, we expected the mean persistence to vary when the parameter was varied. When applied to parameter sets that produced very high chain migration persistence, this allowed us to identify parameters that were most important for sustaining chain migration. This approach provided a systematic and quantitative evaluation of model parameters but did not permit the explicit investigation of spontaneous (or emergent) chain formation from a population of premigratory cells. Chain formation associated with NC cell migration in the gut [31], however, has recently been modelled by another group.

While mean chain migration persistence allowed us to identify critical parameters within a model, it was not practical to use mean chain migration persistence to distinguish between models. This is because timescales in distinct ABMs cannot be explicitly defined, making it difficult to quantitatively conclude, for example, that 40 time steps in the relatively simple ECM Model is fundamentally different from 20 time steps in the far more complex Contact Model. Nor was it practical to directly compare simulated mean persistence values to *in vivo* data. To make accurate persistence comparisons between experimental and simulated data would require the collection of *in vivo* measurements from a very large number of chains over many hours. This would be very expensive in resources and time, making it a nearly impossible experiment to perform with current technology.

**Figure 6.**
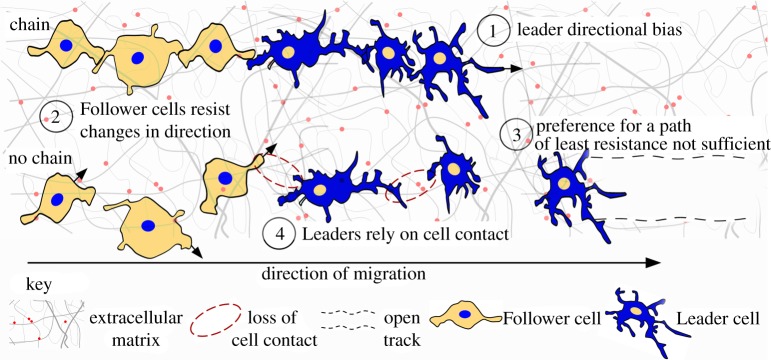
Model characteristics that sustain cell chain migration. Model schematic based on our simulation results that summarize cell behaviours critical for sustaining cell chain migration. The top row of cells portrays a sustained cell chain and the bottom row depicts the breakdown of cell chain migration. (1) Leader directional bias. Leader cells must display a directional bias that is high or moderate (when an open track exists). (2) Follower cells resist changes in direction. Cell chains are sustained when follower cells maintain an initial polarity. (3) Preference for a path of least resistance is not sufficient. Follower cells also need to display directional bias in the presence of an open track. A path of least resistance may promote cell chain migration, as cells move faster towards the target and rely less on cell contact guidance. (4) Leaders rely on cell contact. Chain migration persists when lead cells move as a result of contact, but followers do not move in response to cell contact. Chain migration breaks down when lead cells lose contact with other lead cells and follower cells. Key: the extracellular matrix (ECM) is shown as a fibrous network and includes cell guidance molecules (red dots). An open track designates a path of least resistance in the ECM. The lead cell is portrayed in blue and the follower cell in orange, with the direction of migration from left to right.

From our analyses of simulated chain migration persistence, we developed four major mechanistic conclusions that are summarized in figure 6. First, simulations indicated that, in general, high *Leader* directional bias towards the target promoted chain migration persistence (figures 3*e* and 5*c*; electronic supplementary material, figures S3*b* and S6*c*). Biologically, high directional bias may represent a cell's response to a chemical gradient directing the lead cells towards the target. This is in agreement with recently published experimental data [32,33]. The exception was when an existing track was already present, in which case only moderate *Leader* directional bias could produce high chain persistence (figure 3*e* and electronic supplementary material, figure S6*b*). Mechanistically, modulation in *Leader* directional bias translated into a modulation in *Leader* velocity towards the target in the simulations. Such a modulation *in vivo* may arise owing to a reduced chemical gradient or because lead cells are using contact guidance cues to maintain contact with slower moving trailing cells. It will now be interesting to experimentally investigate this model prediction.

Second, simulations indicated that, in general, high *Follower* directional bias towards the target promoted chain migration (figure 3*d* and electronic supplementary material, figure S3*a*). Biologically, this may also represent a response to a chemical gradient. The only exception was in the Contact and Hybrid Models, where the SID parameter could optionally set the polarized direction of all *Follower* to the target direction at the start of the simulation. If SID was true, the need for high *Follower* directional bias when selecting a new direction was mitigated (figure 5*b*). In addition, chain migration was highest when the direction clocks of *Followers* were unlikely to signal a change of direction (as a consequence of high MDI values; figure 5*e* and electronic supplementary material, figure S4*e*). Together these results suggest that once trailing cells in a chain become polarized in the direction of migration, chains persist longer when trailing cells are resistant to signals to change direction.

Third, simulations indicated that a path of least resistance was not sufficient to drive chain migration persistence but could promote it. The conclusion that a path of least resistance was not sufficient is supported by simulations that indicated *Followers* needed to be completely biased to select the target direction at maximum persistence levels in the ECM Model (figure 3*d*), suggesting a chemotactic mechanism was also necessary. The conclusion that a path of least resistance may promote chain migration, however, is supported by simulations indicating chains moved faster towards the target when a path was already established (compare electronic supplementary material, movies S3–S4 and movies S9–S10) as well as Hybrid Model simulations indicating a preference for a path of least resistance could diminish the importance of contact guidance (electronic supplementary material, figure S7*b*,*c*).

Fourth, simulations indicated that at high mean chain migration persistence levels, *Leaders* relied on their protrusions for guidance information but *Followers* did not (figure 5*d* and electronic supplementary material, figures S3*c*–*f* and S4*b*,*c*). Electronic supplementary material, movies S7, S8 and S11 provide qualitative examples of differences between *Leader* and *Follower* agent protrusion behaviours. It is possible that the difference in preference for contact-mediated guidance exhibited by *Leaders* and *Followers* is due to the rigid phenotypic distinction imposed by the model assumptions. A clear distinction between the hairy phenotype (*Leader* agents) and the polarized phenotype with only a few protrusions (*Follower* agents) has been observed *in vivo* in chick NC cells [26]. Cells in a migratory chain, however, may possess the capacity to be a leader or follower depending on signals each receives. In support of this, data from time-lapse imaging of chick NC cell migration revealed that when a physical barrier placed in front of a wide migratory stream blocked lead NC cells, the trailing NC cells re-routed around the blocked cells and reached the target site [27]. Thus, our observation that *Follower* agents rarely relied on contact-mediated guidance when chain migration persistence was highest, may be due to the restriction that *Follower* agents could only assume a polarized phenotype and suggests that the hairy phenotype may be critical for cell contact guidance. Moreover, the ratio of leader and follower phenotypes in a chain may play a critical role in chain migration persistence. We found that having between 1 and 3, *Leader* agents promoted chain migration persistence in the Contact Model and the ECM Model without a track (electronic supplementary material, table S3 and §5). Additional modelling and experimental analysis are planned to further probe the importance of these phenotypes.

The finding that chain migration persistence was promoted when *Leaders* were likely to maintain cell contact with *Followers* suggests that, mechanistically, lead cells regulate their filopodial dynamics to ensure contact with followers and to communicate position information. Cell communication by contact has previously been suggested as a mechanism for NC cell follow-the-leader behaviour [26], but it was not clear how this communication may be manifested within the migratory population. The formation of thin, cellular bridges *in vivo* that support cytoplasmic transfer of material between neighbouring migratory and dividing neural NC cells, independent of gap junctions, has recently been described [34]. While cytoplasmic transfer has not been investigated in chains, our modelling and experimental work raises the interesting possibility that NC cells communicate spatial information via this process during chain migration.

In light of discoveries related to chemotaxis in cranial NC cell migration [32,33], we are encouraged by model predictions suggesting directional movement of lead and trailing cells is critical to sustain chain migration. The response to a chemical gradient was indirectly included in the ABMs via the LDD and FDD parameters. While recent data suggest that chemotactic response plays a role in migration, it does not seem likely that it can alone account for follow-the-leader chain migration. Our models suggest that chemotaxis along with other processes are involved in the maintenance of follow-the-leader chain migration. Future model refinements will directly include experimental chemotaxis data.

Finally, while our models did not specifically test the contact inhibition of locomotion hypothesis [28], the LMC and FMC parameters in the Contact Model controlled how likely an agent was to move as a result of contact with another agent (a behaviour observed *in vivo* during chick NC cell migration). It is striking that at the highest persistence levels of the Contact Model, the probability that *Followers* moved in response to contact was almost always 0 per cent. Such parameter regimes suggest that contact inhibition of locomotion may be important in the back portion of the chain. While it remains unclear whether contact inhibition of locomotion plays a critical *in vivo* role in chain migration, future model refinements will help to establish the level of significance of this type of mechanism in chain migration persistence.

## 5. Conclusions

Our computational modelling and analyses identified critical parameter conditions that sustain cell chain migration persistence (figure 6). Based on these results, we propose a general model mechanism for NC cell chain migration for the embryonic system on which our model was based. In our proposed model hypothesis (summarized in electronic supplementary material, figure S8), early emerging post-otic NC cells migrate from the NT to target sites in response to guidance signals in the environment, forging paths in the ECM. When later emerging NC cells encounter the migratory pathway, the guidance signals are diminished (either owing to degradation over time or consumption by the early emerging NC cells), resulting in only moderate lead cell directional bias towards the target. By an unidentified mechanism, cells form de novo follow-the-leader migratory chains (possibly spontaneously and see also [31]). Trailing cells become polarized in the direction of the migratory target and lead cells rely on their many protrusions for contact guidance with neighbouring cells and to probe the environment for a reduced chemical signal. Chains that follow an existing trajectory arrive at the target faster than chains that do not.

While chain migration may appear simple, such phenomena are often associated with surprisingly complex cellular behaviours. To fully understand them, unaided biological intuition is not always enough. An integration of experiment and theory will more than likely be required to unravel the complex mechanisms associated with chain migration. The modelling framework we developed provides a foundation for a more detailed investigation of cell chain migration as further cell and molecular data emerge. Our approach also provides a powerful means to test proposed mechanisms of chain migration in other biological systems, such as adult neurogenesis and cancer metastasis.
